# Necrotic enlargement of cone photoreceptor cells and the release of high-mobility group box-1 in retinitis pigmentosa

**DOI:** 10.1038/cddiscovery.2015.58

**Published:** 2015-11-30

**Authors:** Y Murakami, Y Ikeda, S Nakatake, T Tachibana, K Fujiwara, N Yoshida, S Notomi, S Nakao, T Hisatomi, J W Miller, DG Vavvas, KH Sonoda, T Ishibashi

**Affiliations:** 1 Department of Ophthalmology, Graduate School of Medical Sciences, Kyushu University, Fukuoka, Japan; 2 Department of Ophthalmology, Akita University Graduate School of Medicine, Akita, Japan; 3 Department of Ophthalmology, Retina Service, Massachusetts Eye and Ear Infirmary, Harvard Medical School, Boston, MA, USA

## Abstract

Retinitis pigmentosa (RP) refers to a group of inherited retinal degenerations resulting form rod and cone photoreceptor cell death. The rod cell death due to deleterious genetic mutations has been shown to occur mainly through apoptosis, whereas the mechanisms and features of the secondary cone cell death have not been fully elucidated. Our previous study showed that the cone cell death in *rd10* mice, an animal model of RP, involves necrotic features and is partly mediated by the receptor interacting protein kinase. However, the relevancy of necrotic cone cell death in human RP patients remains unknown. In the present study, we showed that dying cone cells in *rd10* mice exhibited cellular enlargement, along with necrotic changes such as cellular swelling and mitochondrial rupture. In human eyes, live imaging of cone cells by adaptive optics scanning laser ophthalmoscopy revealed significantly increased percentages of enlarged cone cells in the RP patients compared with the control subjects. The vitreous of the RP patients contained significantly higher levels of high-mobility group box-1, which is released extracellularly associated with necrotic cell death. These findings suggest that necrotic enlargement of cone cells is involved in the process of cone degeneration, and that necrosis may be a novel target to prevent or delay the loss of cone-mediated central vision in RP.

## Introduction

Retinitis pigmentosa (RP), a major cause of hereditary blindness, is a heterogeneous group of inherited retinal degenerations resulting from rod and cone photoreceptor cell death. Vision loss in RP typically begins with night blindness due to rod cell dysfunction and death, followed by loss of peripheral and central vision because of cone cell death.^1^ Genetic studies have identified mutations in more than 50 genes – most expressed exclusively in rod cells – that are associated with RP.^2,3^ Although it is expected that rod cells die if they harbor the deleterious mutations, it remains unclear why cone cells also die in RP. As the loss of cone-mediated central and peripheral vision is the most debilitating aspect of RP, a better understanding of the mechanisms of cone cell death will be critical to developing novel therapeutics for this currently incurable disease.

Apoptosis and necrosis are two major forms of cell death, defined by their morphological appearance.^4^ Apoptosis is accompanied by the reduction of cellular volume and chromatin condensation, and necrosis is associated with cellular and organelle swelling and plasma membrane rupture. Apoptosis is an active process of cell death, mediated largely through the caspase family of cysteine proteases. Although necrosis was thought to be an unregulated form of cell death, it is now known to have regulated components, such as those involving receptor interacting protein (RIP) kinases.^5,6^

In animal models of RP, rod cell death has been shown to occur mainly through apoptosis.^7,8^ The mode of cone cell death is less characterized. The results of our and other’s previous investigation of mouse models of RP demonstrated that cone cell death is associated with necrotic features such as cytoplasmic and mitochondrial swelling and membrane rupture, and the necrotic cone cell death was substantially suppressed by the genetic or pharmacological inhibition of RIP kinases.^9,10^ These findings indicated that necrotic pathway is involved in cone cell death in RP, at least in part, and may be a novel therapeutic target. However, the relevancy of these findings in human pathology remains unknown.

Adaptive optics (AO) ophthalmoscopy, including AO scanning laser ophthalmoscopy (AO-SLO), allows the noninvasive observation of individual cone cells in living eyes.^11^ With AO-SLO, the cone density in the macula was shown to be decreased and correlate with visual function and photoreceptor layer thickness in RP patients.^12–15^ In the present study, we investigated the possible involvement of necrotic cone cell death in RP patients by examining changes of cone cell size using AO-SLO, and assessing the vitreous levels of necrosis-related proteins.

## Results

### Enlargement of cone cell size in the mouse model of RP accompanies their necrosis-mediated cell death

**Figure 1:**
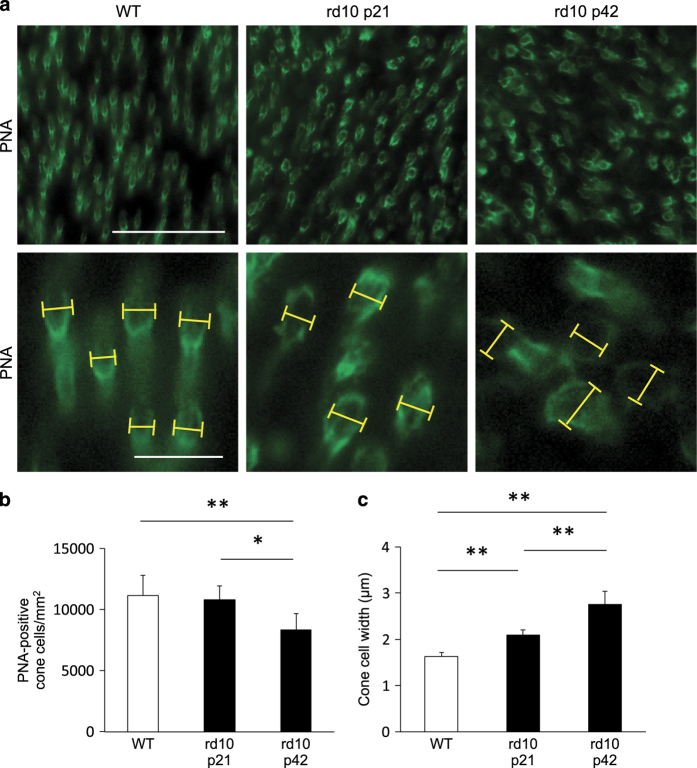
Enlargement of the cone cell size in the mouse model of retinitis pigmentosa. (**a**) Whole-mount staining of the wild-type (WT; left panel) and *rd10* mouse retinas at P21 (middle panel) and P42 (right panel) with PNA. The lower panels are higher magnification of peanut agglutinin lectin (PNA)-positive cone cells. Yellow line: the width of the inner segment of each cone cell. Scale bar, 50 *μ*m (upper panel), 10 *μ*m (lower panel). (**b**, **c**) Quantification of the density (**b**) and width (**c**) of PNA-positive cone cells in WT mice (*n*=6), *rd10* mice at P21 (*n*=6), and *rd10* mice at P42 (*n*=6). *P<0.05, **P<0.01.

*Rd10* mice are an animal model of RP caused by a missense mutation in the *Pde6β* gene.^16^ Mutations in this gene have been found in patients with autosomal recessive RP.^17^ *Rd10* mice develop progressive rod degeneration beginning around postnatal day 18 (P18), and only a slight proportion of rod cells remains at P28; the number of cone cells gradually decreases thereafter. Using biochemical and genetic techniques, we have previously shown that cone cell death occurs via RIP kinase-mediated necrosis.^9^ To investigate changes in cone photoreceptor cell density, morphology, and size, we performed whole-mount immunofluorescence for peanut agglutinin lectin (PNA), which selectively binds to the cone inner and outer segment.^18^ The cone cell density in *rd10* mice was comparable to that in wild-type (WT) mice at P21, whereas it was significantly decreased at P42 in the *rd10* mice (Figures 1a and b; *P*<0.05). We next examined the changes in cellular size during cone degeneration. The width of the inner segment of cone cells was significantly increased in the P21 *rd10* mice, and further enlarged as the loss of cones progressed by day p42 (Figures 1a and c; *P*<0.01). These findings suggest that the enlargement of cone cells occurs in an early phase of cone degeneration in *rd10* mice and is consistent with the necrotic mechanism of cell loss.

**Figure 2:**
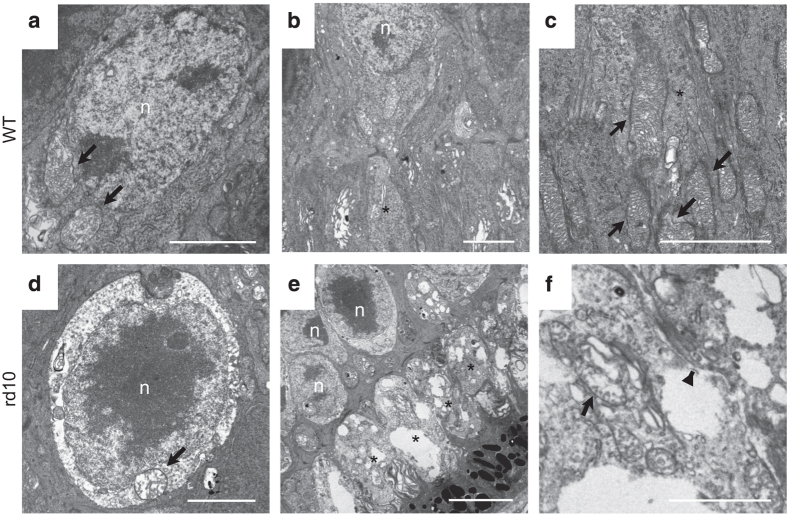
Necrotic changes of cone cells in the mouse model of retinitis pigmentosa. (**a**–**f**) Representative transmission electron microscopy images of the cone photoreceptor cells in wild-type (WT; **a**–**c**, *n*=4) and *rd10* mice (**e**–**f**, *n*=4) at P42. The cone cells in the WT mice contained mitochondria with well-defined lamellar cristae (arrow, **a** and **c**) and cytoplasm with medium electron density (**a–c**). The cone cells in the *rd10* mice showed swelling and vacuolation of the cytoplasm and disruption of the mitochondrial cristae (arrow, **d**). The swollen cone inner segments of *rd10* mice (asterisk, **e**) were associated with plasma membrane rupture (arrowhead, **f**) and mitochondrial disruption (arrow, **f**) n, nucleus; arrow, mitochondria; arrowhead, disruption of the plasma membrane; asterisk, the inner segment of cone cells. Scale bar, 2 *μ*m (**a**–**d**, **f**), 5 *μ*m (**e**).

To further characterize the observed enlargement of cone cells in *rd10* mice, we next performed transmission electron microscopy (TEM) analysis. As we observed in another study,^9^ the cone cells in the p42 *rd10* mice showed swelling of the cytoplasm and disruption of the mitochondrial cristae structure (Figure 2d). The inner segments of the cone cells of the *rd10* mice were substantially swollen, accompanied by the vacuolation of the cytoplasm, plasma membrane discontinuation, and mitochondrial rupture, which are the characteristics of necrosis (Figures 2e and f). These findings demonstrate that necrotic cellular enlargement occurs during cone degeneration in *rd10* mice.

### AO-SLO imaging demonstrate enlarged cone cell size in the RP patients

**Figure 3:**
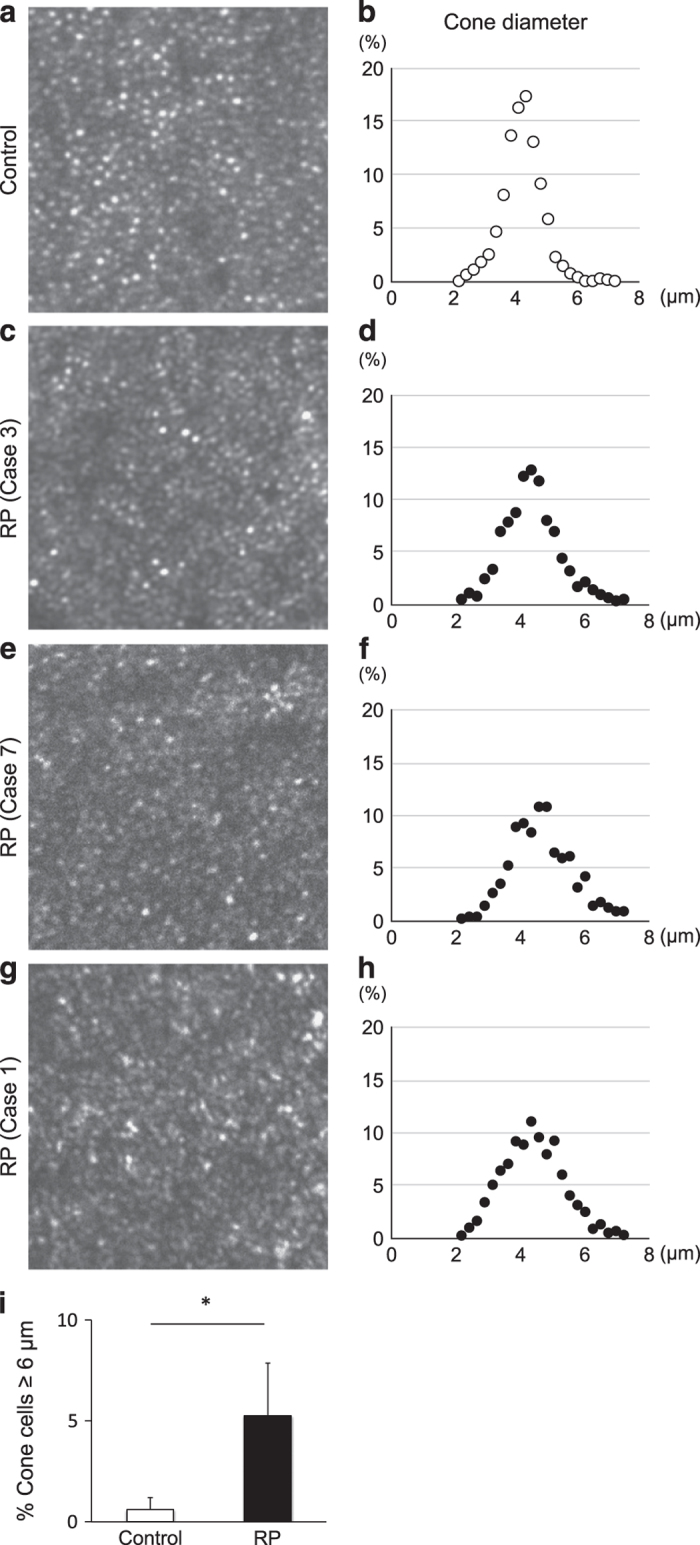
Cone size analysis in human patients with retinitis pigmentosa. (**a**–**h**) Representative adaptive optics scanning laser ophthalmoscopy (AO-SLO) images (**a**, **c**, **e**, **g**) and the spot diameter analysis (**b**, **d**, **f**, **h**) of the retina at 1.0 mm from the foveal center in a control subject (**a**, **b**) and retinitis pigmentosa (RP) patients with variable disease severity (**c–h**) The RP patients showed a decreased percentage of bright spots within 3.5–5.0 *μ*m, and an increased percentage of large spots with ⩾6.0-*μ*m diameter. The enlargement of bright spots was observed in a case with an intact ellipsoid zone (EZ) (**d**), as well as in cases with abnormal EZ (**f**) and absent EZ (**h**) at 1.0 mm eccentricity from the fovea. (**i**) Quantification of the percentage of bright spots with ⩾6.0-*μ*m diameter in the control subjects (*n*=7) and RP patients (*n*=10). The RP patients had significantly higher percentages of enlarged bright spots compared with the controls (5.3±2.5 *versus* 0.6±0.6%) *P=0.0004.

We next analyzed cone cell size in human RP patients using AO-SLO images. As shown in Table 1, there were no significant differences in age, gender distribution, or axial length of the eye between the 10 RP patients and the 7 healthy controls. The cone mosaic images were obtained in the retinal areas at 1.0 mm from the foveal center, and the diameter of each bright spot was calculated. Figure 3 presents the representative cone mosaic images and plots of the spot diameter in a control subject and RP patients with variable disease severity (Table 2, Supplementary Figures S1). The bright spot diameters in the control subjects were mostly within the range 3.5–5.0 *μ*m, and the spots with ⩾6.0-*μ*m diameters were rarely observed (Figure 3b). In contrast, the RP patients showed decreased percentages of the bright spots within 3.5–5.0 *μ*m, and they had a small but significant population of large spots with ⩾6.0-*μ*m diameters (Figures 3d, f and h). The enlargement of bright spots were observed in a case with an intact ellipsoid zone (EZ; Figure 3d, Supplementary Figure S1) as well as in cases with abnormal (discontinuous) EZ (Figure 3f, Supplementary Figure S2) and absent EZ (Figure 3h, Supplementary Figure S3) at the area examined by AO-SLO.

The quantitative analysis confirmed that the RP patients had significantly higher percentages of enlarged bright spots with ⩾6.0 *μ*m compared with the control subjects (5.3±2.5 *versus* 0.6±0.6%, *P*=0.0004, Figure 3i). Although the RP patients with absent or abnormal (discontinuous) EZ had relatively higher percentages of enlarged bright spots (6.7±2.3%), two RP patients with an intact EZ still exhibited an increased proportion of enlarged bright spots (5.7% in case 3 and 3.0% in case 8; Table 2, Supplementary Figure S4). Only one RP patient showed a normal distribution of spot size (case 9).

### Increased HMGB1 levels in the vitreous of the RP patients

**Figure 4:**
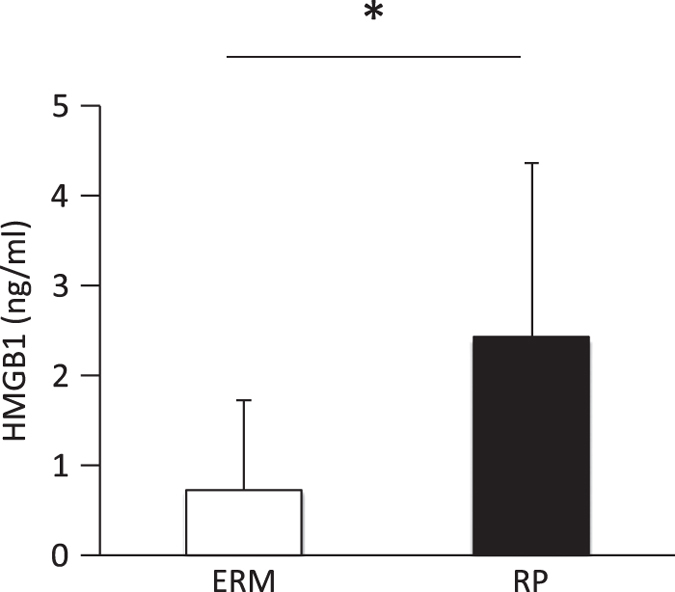
Increased levels of high-mobility group box-1 in the vitreous of patients with retinitis pigmentosa. Protein levels of released high-mobility group box-1 (HMGB1) in the vitreous of control patients with epiretinal membrane (ERM; *n*=10) and patients with ERM secondary to retinitis pigmentosa (*n*=10). *P=0.0312.

High-mobility group box-1 (HMGB1) is an intracellular DNA-binding protein that regulates transcription activity; however, it is released into the extracellular space under stressed conditions that cause necrosis, and it mediates inflammatory signals.^19–21^ To further investigate the involvement of necrotic cell death in RP, we examined the changes of HMGB1 release into the vitreous of the RP patients. The baseline characteristics of RP and control patients were shown in Table 3. The HMGB1 levels were significantly higher in 10 patients with RP (2.4±1.9 ng/ml) compared with 10 patients in our control (0.7±1.0 ng/ml, *P*=0.0312; Figure 4).

## Discussion

In the present study, we demonstrated that the cone cells in *rd10* mice show cellular enlargement during cone degeneration, and are associated with necrotic features such as cytoplasmic and organelle swelling and membrane rupture. The involvement of necrosis in cone cell death is also reported in mice deficient for interphotoreceptor retinoid-binding protein (Irbp), another model of RP.^10^ With the use of AO-SLO, our study showed that a significant number of cone cells exhibit enlarged spot sizes in the macula of RP patients. In addition, the vitreous levels of HMGB1 were increased in RP patients. Taken together with the results from experimental studies, these findings may suggest the involvement of necrotic cone cell death in the pathology of human RP. Consistent with this idea, previous histological studies of postmortem RP patients’ eyes demonstrated the necrotic changes of cone cells such as swollen cytoplasm and disrupted plasma membrane.^22^

Although the latest generation of AO ophthalmoscopy is able to resolve both rod and cone photoreceptor cells,^23,24^ the resolution limits of our confocal AO-SLO system did not allow visualization of rod cells because of their small cell size. In this study, we showed that the bright spots in AO-SLO imaging, the signal of which is known to derive largely from the cone outer segments,^25^ was approximately 3.5–5.0 *μ*m in diameter at 1.0 mm eccentricity from the fovea in control subjects. These spot sizes appear to be smaller than the diameter of cone inner segments at the same eccentricity in the postmortem human eyes (6.0–7.5 *μ*m).^26^ This may be due to the differences in cell compartment visualized by each method. These findings are consistent with a recent study showing that the cone inner segment structure obtained by non-confocal split detector AO-SLO is larger than the corresponding bright spot in confocal AO-SLO images.^27^

Previous AO imaging studies of RP patient eyes have shown that the cone bright spots become obscure or absent in areas with severe retinal degeneration, probably due to the loss of cone cells or the disruption of the outer segment structure. Because the blurring of cone mosaic image may lead to incorrect measurement of the spot size, we obtained AO-SLO images from RP patients who retained good visual acuity. Our study showed that the bright spot sizes in the RP patients were largely comparable to those of the controls, suggesting that the quality of cone images may be appropriate for the analysis. In addition, we found a small but significant population of cone cells with increased spot sizes in RP patients. The enlargement of cone image was observed even in patients with intact EZ, suggesting that these findings may reflect the early morphological changes of cone cells during cone degeneration in RP. The question of whether the enlarged bright spots will finally be lost and become dark areas in AO-SLO warrants further longitudinal investigation.

Intracellular contents released from dying or dead cells act as damage-associated molecular patterns (DAMPs) to promote inflammatory responses and tissue injury.^28^ HMGB1 is one of the best-characterized DAMPs released from necrotic cells.^19,20^ In the vitreous of patients with retinal detachment, which involves not only apoptotic cell death but also necrotic photoreceptor cell death, the levels of extracellular HMGB1 are increased and correlate with chemokine levels.^29^ Consistent with these findings, we observed that the HMGB1 levels in the vitreous of the RP patients were significantly elevated compared with those in the nondegenerative controls, supporting the idea that necrotic cell death is implicated in the degenerative process of RP. We recently observed that the vitreous of RP patients contains substantial amounts of inflammatory cytokines and chemokines, and that intraocular inflammation measured by slit-lamp or laser flaremeter is inversely associated with central visual function.^30,31^ Although the relationship between cell death and inflammation needs to be further investigated, DAMPs released from necrotic cells may enhance inflammation and retinal degeneration in RP.

Although the precise cell source of HMGB1 in RP patient eyes remains unknown, the histological findings of animal models and human patients with RP suggest that necrotic cone cells may be an important source of HMGB1. It has been shown that the rod cell death caused by genetic mutations occurs mainly through apoptosis in several models of RP.^7,8^ Consistent with these findings, our previous study showed that RIP kinase inhibition suppresses necrotic cone but not apoptotic rod cell death in *rd10* mice.^9^ In contrast, Sato *et al.* ^10^ recently reported that rod cell death is partly mediated by the RIP kinase-dependent pathway in *Irbp*-deficient mice. Although the mutations in *IRBP* are infrequent in RP patients,^32^ these findings suggest that necrotic rod cell death may exist in a subset of RP. However, because the RP patients who underwent vitrectomy in our study were at a relatively advanced stage of the disease associated with severe rod degeneration, it is unlikely that there remain large amounts of rod-derived DAMPs in the vitreous of these patients.

A limitation of our study is the lack of information regarding the genetic backgrounds of the RP patients. However, the variable inheritance mode and clinical course among the patients suggest that the enlargement of cone cells may be associated with the heterogeneous condition of RP. Another limitation is the different patient backgrounds between patients involved in the AO-SLO study and patients analyzed for the vitreous HMGB1. Because there is limited opportunity to obtain vitreous samples from patients in early stages of RP, we were unable to address the earlier HMGB1 changes in the vitreous of RP patients. Nevertheless, taken together with the results from experimental studies,^9^ these findings suggest that necrotic cell death may be involved in diverse phases of cone degeneration in RP. Further studies to investigate the relationships among genes, disease courses, and cone cell changes will be important for the elucidation of cone cell mechanisms in RP.

In conclusion, the results of the present study demonstrated that in a mouse model of RP, dying cone cells show necrotic morphology such as cellular and organelle swelling and rupture. The enlargement of cone cells was also observed in the macula of RP patients in our AO-SLO analysis, and the extracellular release of HMGB1, a necrosis-related DAMP, was increased in the vitreous of the RP patients. Taken together, these findings indicate that necrotic cone cell death may underlie the pathogenesis of RP, and suggest necrosis-related molecular pathways as novel targets for preventing or delaying the loss of cone-mediated central vision in RP.

## Materials and methods

### Ethics statement

This clinical study was approved by the Institutional Review Board of the Kyushu University Hospital (Fukuoka, Japan), and was conducted in accordance with the tenets of the Declaration of Helsinki Principles on biomedical research involving human subjects. Informed consent was obtained from all subjects after a thorough explanation of the nature of the study and its possible consequences.

All animal experiments adhered to the statement of the Association for Research in Vision and Ophthalmology, and the protocols were approved by the Committee for Animals, Recombinant DNA, and Infectious Pathogens Experiments at Kyushu University.

### Animals

B6.CXB1-*Pde6*^*rd10*^/J (*rd10*) and WT C57BL/6J mice were purchased from The Jackson Laboratories (Bar Harbor, ME, USA).

### PNA staining

The mouse eyes were enucleated and fixed in 4% paraformaldehyde (PFA) for 1 h. After the eyes were washed with phosphate-buffered saline (PBS), the neuroretinas were incubated with blocking buffer (10% nonfat dried milk and 0.3% Triton X in PBS) for 1 h, and then incubated with fluorescein isothiocyanate-conjugated PNA (1 : 100; Sigma-Aldrich, St. Louis, MO, USA) at 4 °C overnight. The neuroretinas were examined with a microscope (Observer.Z1, Zeiss, Jena, Germany).

The number of cones in each neuroretina was determined as described.^9^ The retinal areas of 0.12 mm^2^ located 0.5 mm superior, inferior, temporal, and nasal to the center of the optic nerve were photographed, and the number of PNA-positive cells in the four regions were counted using ImageJ software (National Institute of Health, Bethesda, MD, USA) and averaged. The width of the inner segment of PNA-positive cells was measured in each retinal area using ImageJ software, and those of over 150 cells per eye were averaged. The retinal samples were assigned numbers and letters, and the conditions were masked from the observers.

### Transmission electron microscopy

The mouse eyes were enucleated, and the posterior segments were fixed in 2.5% glutaraldehyde and 2% PFA in 0.1 M cacodylate buffer with 0.08 M CaCl_2_ at 4 °C. The sections of retinas, retinal pigment epithelium, and choroid complexes were post-fixed for 1.5 h in 2% aqueous OsO_4_, dehydrated in ethanol and water, and embedded in EPON medium. Ultrathin sections were cut from blocks and stained with saturated aqueous uranyl acetate and Sato’s lead stain. The specimens were observed with TEM (Hitachi H-7650, Hitachi High-Technologies, Tokyo, Japan).

### Patients

Ten patients with the diagnosis of typical RP and seven control subjects without ocular diseases were included in the AO-SLO study. Patients were recruited from the Kyushu University Hospital. The baseline characteristics of these subjects are shown in Tables 1 and 2.

The vitreous fluids were obtained from a different group of 10 RP patients who underwent vitrectomy for ERM removal. Those from 10 idiopatic ERM patients were used as controls. All patients received surgery at Kyushu University Hospital. The baseline characteristics of these subjects are shown in Table 3.

The diagnosis of typical RP was based on a patient’s history of night blindness, visual field constriction and/or ring scotoma, and markedly reduced or nonrecordable a- and b-wave amplitudes on electroretinography testing, in addition to ophthalmoscopic findings (e.g., bone spicule-like pigment clumping in the mid-peripheral and peripheral retina and attenuation of retinal vessels). Excluded from the study were patients with cone–rod or cone dystrophy, Bietti crystalline retinopathy, retinal inflammatory diseases, or autoimmune paraneoplastic retinopathy.

### Clinical examinations

The best-corrected visual acuity (VA) was measured with a standard Japanese decimal VA chart and converted to the logarithm of the minimum angle of resolution (logMAR) units. Automated static perimetry tests were performed by the Humphrey Field Analyzer (Humphrey Instruments, San Leandro, CA, USA) using the central 10–2 Swedish Interactive Thresholding Algorithm Standard Program. The lens was corrected as appropriate for the test distance. Visual field testing was repeated if the test reliability was not satisfactory (fixation loss >20%, false positive >15%, or false negative >33%). The averaged foveal sensitivity at the central four points was analyzed as described.^33,34^ The test was examined twice, and the better result was used for the analysis in order to reduce the learning effect. Axial length was measured using IOLMaster non-contact optical device (Carl Zeiss, Dublin, CA, USA). Fundus autofluorescence and spectral-domain optical coherence tomography images were obtained with the Spectralis HRA-OCT (Heidelberg Engineering, Heidelberg, Germany). The appearance of EZ at 1.0 mm eccentricity from the fovea was classified into three categories: absent (EZ was not visible), abnormal (EZ was visible but disrupted or discontinuous), and normal, as described.^35^ These examinations were performed on the same visit day for each subject.

### Adaptive optics scanning laser ophthalmoscopy

High-resolution images of cone photoreceptor cells in the macula were obtained with the Canon AO-SLO system (Canon Inc., Tokyo, Japan) as described.^14,36^ Images focused on the photoreceptor layer were recorded in the areas of 340×340 *μ*m located 1.0 mm superior, inferior, temporal, and nasal to the central fovea. Thirty-two images were acquired and averaged at each region. Each cone cell in the AO-SLO image was identified using a modified version of the automated cone labeling program of Li and Roorda.^37^ This program automatically applied low pass filters to the cone mosaic image, with selected cutoff frequency according to the retinal eccentricity. The images with automated cone labeling were examined by a masked observer (SN), and cone cells that were visible but had not been labeled by the program, were manually identified in the analyzer software.

The spot size of each cone cell was obtained using scale selection method proposed by Lindberg.^38^ At each cone cell location identified, local extrema over sigmas (*σ*max) of scale-normalized laplacian of gaussian was automatically calculated. The spot size was calculated as follows: 2×√2*σ*max (pixel)×0.85 (*μ*m/pixel) (Supplementary Figure S5). Over 500 spots per eye were analyzed.

### ELISA

The protein levels of HMGB1 in the vitreous fluids were determined with an ELISA kit for HMGB1 (Shinotest Corp., Tokyo, Japan) according to the manufacturer’s instructions.

### Statistical analysis

The data are presented as the arithmetic mean±S.D. Statistical differences in the mean values between the groups were analyzed by independent-sample *t*-tests, and the differences of frequency were tested by *χ* ^2^-tests. We performed a multiple group comparison by an analysis of variance followed by Tukey–Kramer adjustments. All of the statistical analyses were performed with SAS software (version 9.3, SAS Institute, Cary, NC, USA). Two-sided values of *P*<0.05 were considered significant.

## Figures and Tables

**Table 1:** The baseline characteristics of the retinitis pigmentosa patients and controls in the adaptive optics scanning laser ophthalmoscopy study

	*Controls*	*Patients with RP*	**P**-*value*
	(**n** *=7)*	(**n** *=10)*	
Age	27.1±3.8 (23–35)	31.5±5.7 (23–40)	0.5284
Gender (males : females)	3:4	2:8	0.1168
Axial length (mm)	24.8±1.5	24.4±1.4	0.5284
VA (logMAR)		−0.01±0.05	
MD value (dB)		−12.5±1.1	
Foveal sensitivity (dB)		32.0±4.4	

Abbreviations: dB, decibel; logMAR, logarithm of the minimum angle of resolution; MD, mean deviation; RP, retinitis pigmentosa; VA, visual acuity.

Data are expressed as mean±S.D.

Ocular characteristics are derived from the right eyes

**Table 2:** Characteristics of the retinitis pigmentosa patients in the adaptive optics scanning laser ophthalmoscopy study

*Case*	*Age*	*Sex*	*Inheritance mode*	*AL (mm)*	*VA (logMAR)*	*MD (dB)*	*FS (dB)*	*EZ at 1 mm from the fovea*	*Cone cells *⩾*6 * **μ** *m (%)*
1	24	M	S	22.3	0.10	−17.47	24.25	Disappeared	3.6
2	24	F	S	24.1	0.00	−15.2	33	Abnormal	6.0
3	31	F	AR	25.2	−0.08	−4.68	35	Normal	5.7
4	40	F	S	23.5	0.00	−11.64	26	Disappeared	4.3
5	31	M	AR	25.2	0.00	−25.59	30.5	Abnormal	4.6
6	23	F	S	25.3	0.22	−23.23	29.75	Disappeared	8.2
7	35	F	S	22.0	−0.08	−17.71	33.5	Abnormal	10.3
8	36	F	S	25.1	−0.08	−1.16	36.2	Normal	3.0
9	35	F	AD	26.5	−0.18	1.67	37.5	Normal	0.5
10	36	F	AD	24.5	0.00	−10.11	34	Abnormal	6.4

Abbreviations: AD, autosomal dominant; AL, axial length; AR, autosomal recessive; dB, decibel; EZ, ellipsoid zone; F, female; FS, foveal sensitivity; logMAR, logarithm of the minimum angle of resolution; M, male; MD, mean deviation; S, spontaneous; VA, visual acuity.

Ocular characteristics are derived from the right eyes

**Table 3:** The baseline characteristics of the retinitis pigmentosa patients and controls in the ELISA

	*Patients with idiopathic ERM (* **n** *=10)*	*Patients with ERM secondary to RP (* **n** *=10)*	**P-** *value*
Age	70.1±5.8 (60–81)	58.9±14.3 (31–73)	0.049
Gender (males : females)	4:6	3:7	0.640
VA (logMAR)	0.21±0.28	0.82±0.53	0.006
MD value (dB)	−1.3±2.8	−18.6±10.5	0.001
Foveal sensitivity (dB)	31.8±3.4	17.3±11.0	0.003

Abbreviations: dB, decibel; ERM, epiretinal membrane; LogMAR, logarithm of the minimum angle of resolution; MD, mean deviation; RP, retinitis pigmentosa; VA, visual acuity.

Data are expressed as mean±S.D.
