# Supplementary figures and images for: Necrotic enlargement of cone photoreceptor cells and the release of high-mobility group box-1 in retinitis pigmentosa
Source: Cell Death Discov. 2015 Nov 30;1:15058–. doi: 10.1038/cddiscovery.2015.58 (PMC4979449; doi:10.1038/cddiscovery.2015.58)

**A**

**B**

**C**

**D**

|    |    |    |    |    |  |    |    |    |    |
|----|----|----|----|----|--|----|----|----|----|
|    |    |    |    | 18 |  | 14 |    |    |    |
|    |    | 17 | 18 | 25 |  | 17 | 19 | 20 |    |
|    | 19 | 19 | 21 | 24 |  | 25 | 25 | 27 | 23 |
|    | 22 | 32 | 33 | 31 |  | 35 | 35 | 27 | 23 |
| 24 | 29 | 32 | 34 | 33 |  | 35 | 36 | 32 | 27 |
| 22 | 25 | 31 | 35 | 32 |  | 33 | 36 | 36 | 32 |
|    | 26 | 33 | 36 | 34 |  | 35 | 36 | 36 | 32 |
|    | 24 | 32 | 34 | 33 |  | 33 | 34 | 35 | 34 |
|    |    | 34 | 33 | 32 |  | 33 | 35 | 33 |    |
|    |    |    |    |    |  | 27 | 30 |    |    |

**E**

**F**

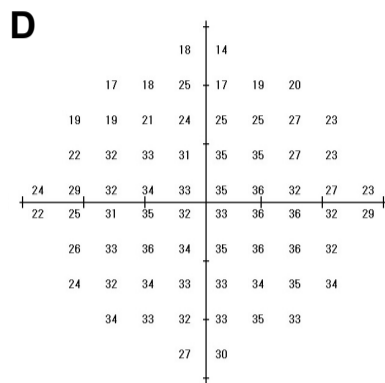

Figure S2

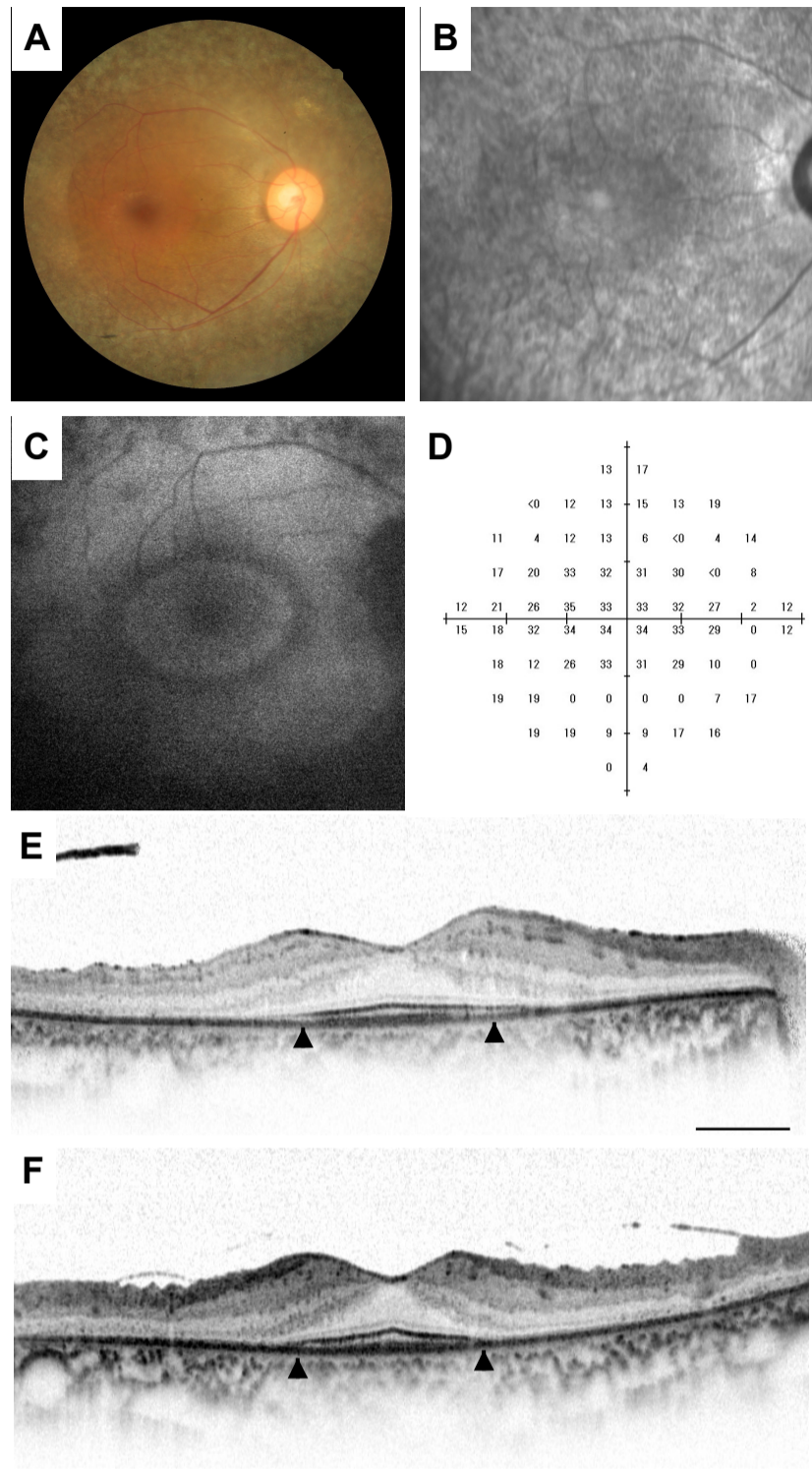

**A**

**B**

**C**

**D**

**E**

**F**

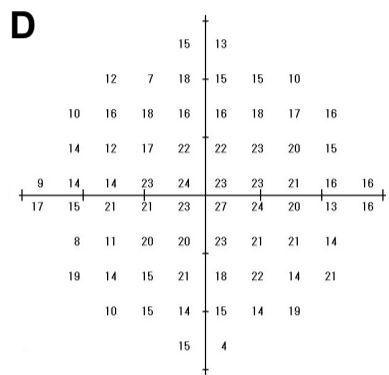

## Figure S4

**A**

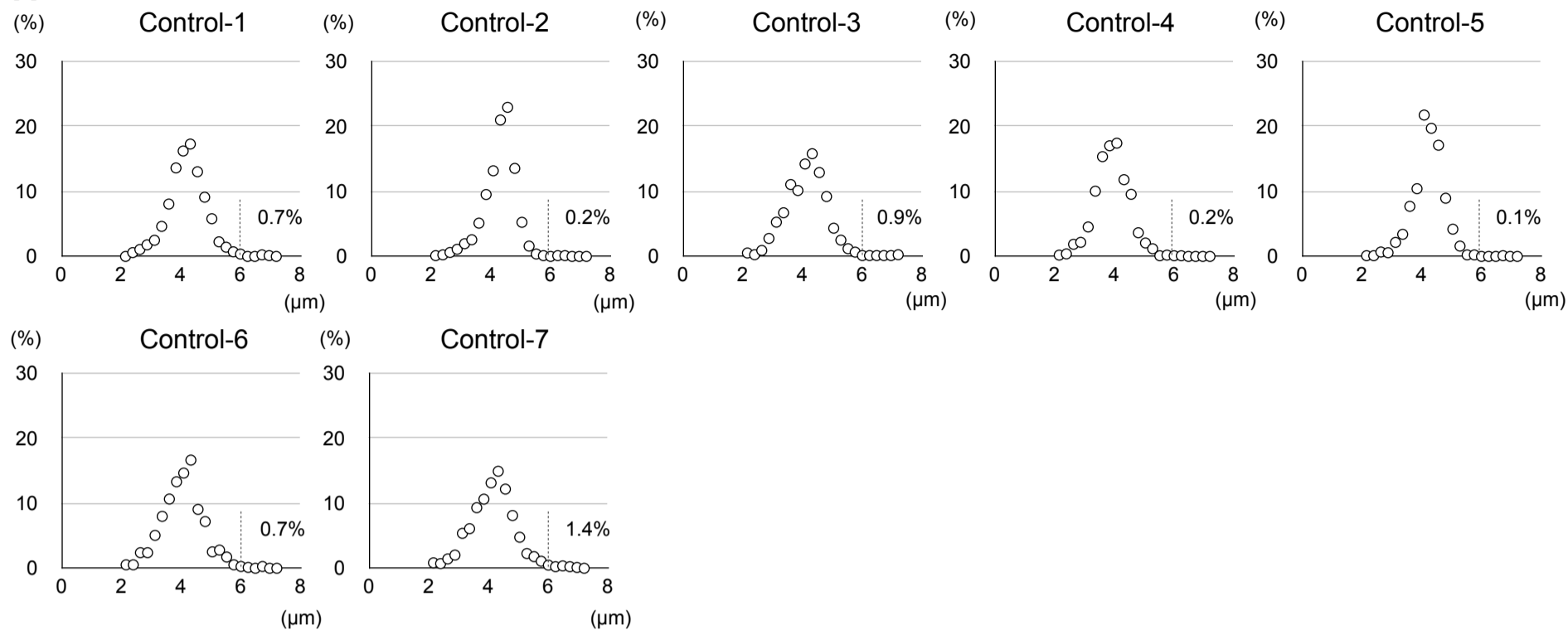

**B**

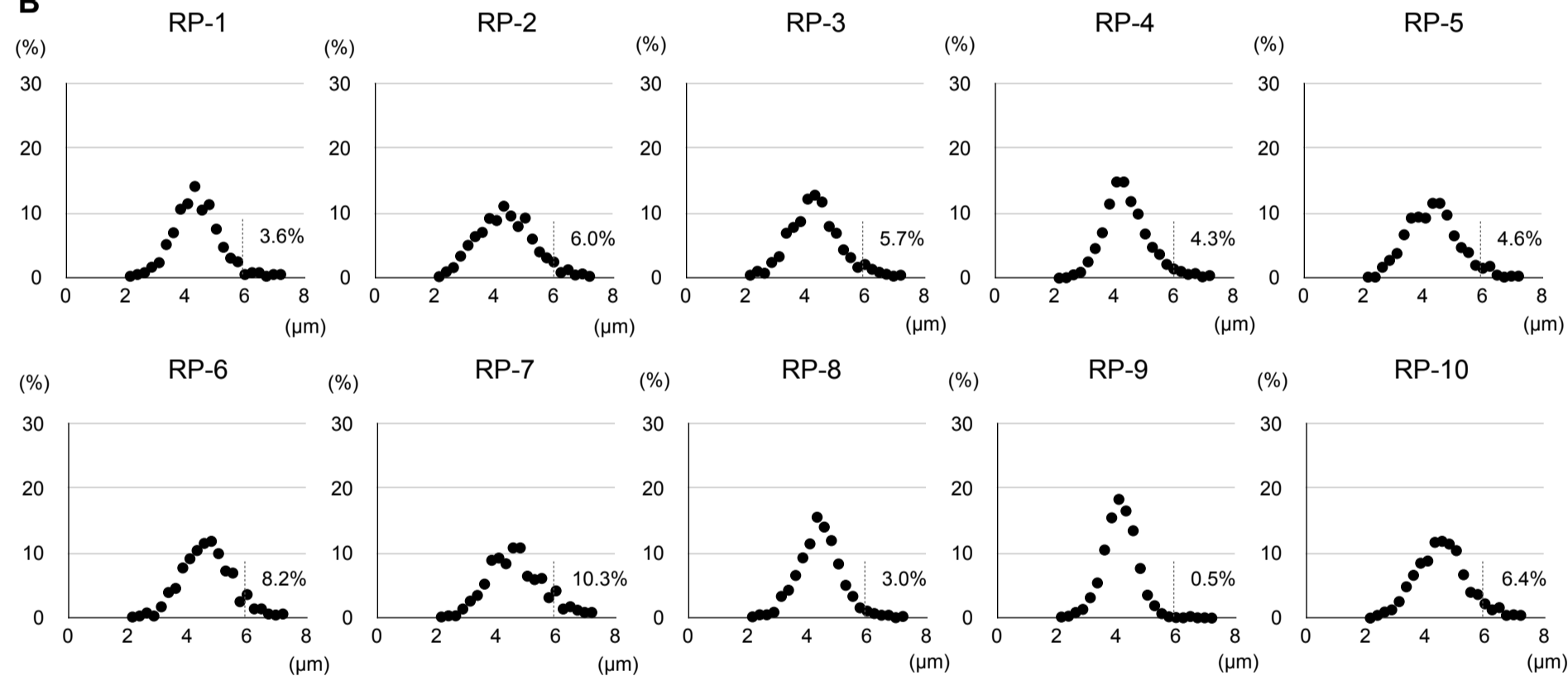

Figure S5

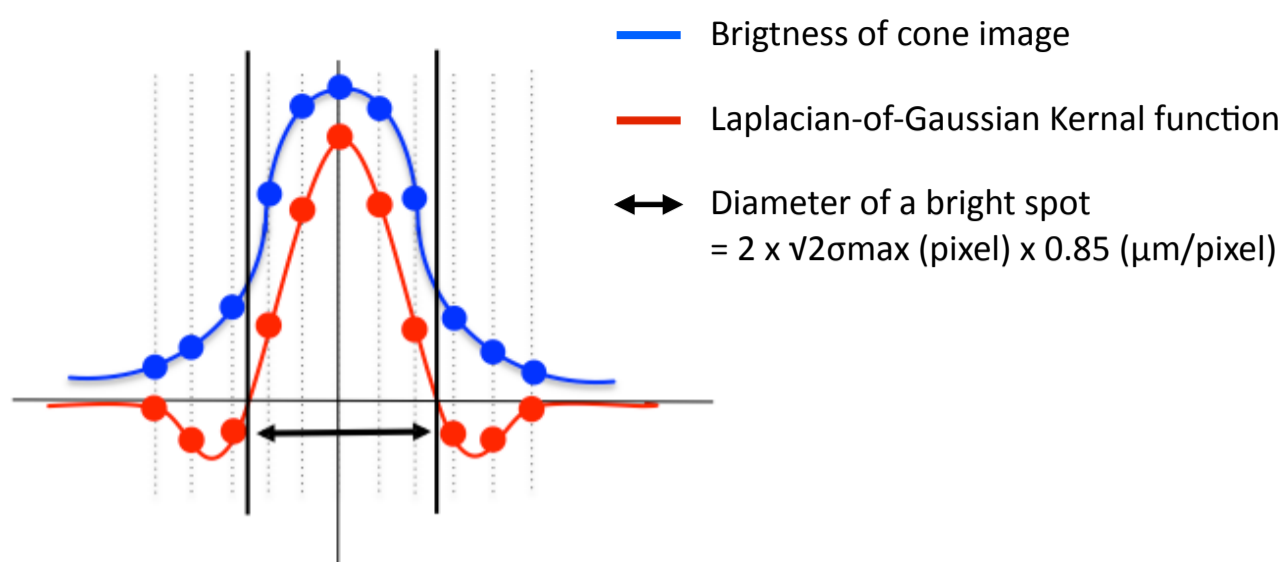

Supplement: Supplementary Figures [file cddiscovery201558-s1.pdf]
