# Supplementary material for: Necrotic enlargement of cone photoreceptor cells and the release of high-mobility group box-1 in retinitis pigmentosa
Source: Cell Death Discov. 2015 Nov 30;1:15058–. doi: 10.1038/cddiscovery.2015.58 (PMC4979449; doi:10.1038/cddiscovery.2015.58)
Supplement: Supplementary Figure Legends [file cddiscovery201558-s2.doc]

**Supplementary Figure Legends**

**Fig. S1. Clinical characteristics of a patient with retinitis pigmentosa (case 3).**

A 31-year-old woman with retinitis pigmentosa. A: Fundus photograph showing the attenuation of retinal vessels and the mild atrophy of retinal pigment epithelium in the perivascular and the mid-periphery. The macular region was preserved with a good foveal reflex. B,C: Infrared and fundus autofluorescence (FAF) images. FAF image showing a perifoveal ring of hyperfluorescence. D: The result of Humphrey Field Analyzer tests using the central 10-2 Swedish Interactive Thresholding Algorithm Standard Program. The visual sensitivity within 10-degree visual field was mostly preserved. E,F: Spectral-domain optical coherence tomography images of a horizontal (E) and vertical (F) line scan through the fovea. Note that the ellipsoid zone was intact at 1.0 mm from the foveal center (arrowheads). Scale, 1 mm.

**Fig. S2. Clinical characteristics of a patient with retinitis pigmentosa (case 7).**

A 35-year-old woman with retinitis pigmentosa. A: Fundus photograph showing the attenuation of retinal vessels and moderate atrophy of retinal pigment epithelium extending to around the fovea. B,C: Infrared and fundus autofluorescence (FAF) images. FAF image showing a parafoveal hyperfluorescent ring surrounded by a hypofluorescent ring in the macula. D: The result of Humphrey Field Analyzer tests using the central 10-2 Swedish Interactive Thresholding Algorithm Standard Program. The visual sensitivity in the central 12 points was relatively preserved. E,F: Spectral-domain optical coherence tomography images of a horizontal (E) and vertical (F) line scan through the fovea. Note that the ellipsoid zone was discontinuous at 1.0 mm from the foveal center (arrowheads). Scale, 1 mm.

**Fig. S3. Clinical characteristics of a patient with retinitis pigmentosa (case 1).**

A 24-year-old man with retinitis pigmentosa. A: Fundus photograph showing the attenuation of retinal vessels and moderate atrophy of retinal pigment epithelium extending to within the fovea. B,C: Infrared and fundus autofluorescence (FAF) images. FAF image showing a central hyperfluorescence surrounded by a hypofluorescent ring in the macula. D: The result of Humphrey Field Analyzer tests using the central 10-2 Swedish Interactive Thresholding Algorithm Standard Program. The visual sensitivity was decreased at all the points examined. E,F: Spectral-domain optical coherence tomography images of a horizontal (E) and vertical (F) line scan through the fovea. Note that the ellipsoid zone was absent at 1.0 mm from the foveal center (arrowheads). Scale, 1 mm.

**Fig. S4. Cone size analysis of adaptive optics scanning laser ophthalmoscopy images.**

Plots of the spot diameter from 7 control subjects (A) and 10 patients with retinitis pigmentosa (B). The numbers in the graph indicate the percentage of bright spots with ≥ 6.0-μm diameter.

**Fig. S5. Method for the measurement of spot diameter in adaptive optics scanning laser ophthalmoscopy.**

The brightness of each cone cell image (blue line) was convoluted with the Laplacian-of-Gaussian kernel (red line). The maximum of the convoluted value with changing σ, a standard deviation of the Laplacian-of-Gaussian function, reflects the characteristic size of the corresponding image structure. The spot diameter (arrow) was calculated as follows: 2 x √2σmax (pixel) x 0.85 (μm/pixel).
